# Supplementary material for: Sex biased effect of acute heat shock on the antioxidant system of non-native round goby Neogobius melanostomus
Source: PLoS One. 2021 Dec 16;16(12):e0260641. doi: 10.1371/journal.pone.0260641 (PMC8675664; doi:10.1371/journal.pone.0260641)
Supplement: Attachment — Submitted filename: reviewer Comments.docx [file pone.0260641.s001.docx]

**Reviewer Comments**

**Manuscript number:** PONE-D-21-22718

**Article type:** Research Article

**Full title:** Sex biased effect of acute heat shock on the antioxidant system of non native Round Goby, *Neogobiusmelanostomus*

**Short title:** Sex biased response of Round Goby to acute heat shock

**Corresponding author:** DagmaraBlonska

University of Lodz, Faculty of biology and environmental protection, Lodz, Poland

**Keyword:** Oxidative stress, Invasive species, Thermal stress, Reproductive effort, Gender differences.

**Reviewer Comments**

This paper investigates an interesting aspects of redox physiology regarding sex biased effect of acute heat shock in Round Goby.However its current state leaves much to be desired. Antioxidant status in females has been reported to be significantly lower than the males which are not as per the previous findings in many animals. Reproductive investment by males has been told to be more than the female which needs more evidence in its support. Some graphs need symbols clarifying the difference between different groups withappropriate level of significance. While the paper is well written, it needs more explanations about the findings citing more references.

**Major concerns:**

1. Authors have mentioned “Male reproductive investment and likelihood of only single spawningperiod in their lifetime does not enable them to develop efficient antioxidant defence”, in the abstract section. However from the graph it seems males to be with higher level of GSH, Catalase and TAC in control condition than the female counter parts. This contradiction should be explained.
2. It has been reported in many animals regarding higher antioxidant levels in females than males. Even sex hormone estrogen shows its antioxidant activity. Here authors have reported an opposite result which should be explained and should be supported with more evidences considering other antioxidant enzymes and non enzymatic antioxidant.
3. Regarding reproductive investment, it was told that the female Round Goby’s reproductive investment is less than the male. It should also be explained with appropriate reason and citation. Usually females invest a lot than the male for reproduction.
4. Authors have shown in the graph regarding higher responses in changes of antioxidant level by male Goby than the female. However no explanation regarding increase, decrease or coming down to the control level have been given with respect to pro-oxidant (MDA) and antioxidant after heat treatment.
5. In Discussion section (246-248) it was told ..........”It is possible that via the transport network GSH demands in muscle were met by liver supply, which can be evidenced in males by the increased value of GSH in 6h treatment in muscle with a simultaneous decrease in liver”. However graph showing GSH data is different i.e. GSH in 6h treatment in muscle of male is decreasing and GSH in 6h treatment in liver of male is increasing. Likewise in the line 253-256 it was told that increase in GSH level after 12h treatment, Coincides with a decrease in MDA levels (but not significant). However graph shows no decrease in MDA level in muscle tissue after 12h treatment.
6. Results as represented in graphs have not been explained considering variations in data due to heat treatment and sex biasedness.

**Minor Concerns**

1. Abbreviations used in graphs for Significantdifferences among different group should be clearly mentioned in the caption.
